# Case report: Real-world experience using a personalized cancer-specific circulating tumor DNA assay in different metastatic melanoma scenarios

**DOI:** 10.3389/fonc.2022.978996

**Published:** 2022-11-17

**Authors:** Karam Khaddour, Alice Zhou, Omar H. Butt, Griffin Budde, Allyson Koyen Malashevich, George Ansstas

**Affiliations:** ^1^ Department of Medicine, Division of Oncology, Washington University School of Medicine, Saint Louis, MO, United States; ^2^ Department of Medicine, Division of Hematology and Oncology, University of Illinois at Chicago, Chicago, IL, United States; ^3^ Department of Neurology, Washington University School of Medicine, Saint Louis, MO, United States; ^4^ Oncology, Natera, Inc, Austin, TX, United States

**Keywords:** melanoma, metastatic disease, ctDNA, molecular residual disease, minimal residual disease

## Abstract

Circulating-tumor DNA (ctDNA) has emerged as an important biomarker for monitoring disease status in cancer patients. Different ctDNA testing platforms have shown promising results in the early detection of disease, monitoring response to treatment, and prognostication in metastatic melanoma. However, several challenges exist, including the reduced shedding of ctDNA into the bloodstream in the metastatic setting, differences in sensitivity among various ctDNA assays, and the inherent inability to distinguish tumor-specific mutations from other mutations that are not related to the cancer of interest. Using a ctDNA assay that is designed to detect multiple single-nucleotide variants (SNVs) that are specific to the tumor itself may allow for more accurate monitoring of disease status in metastatic melanoma. In this case series, we describe a real-world experience using a personalized, tumor-informed ctDNA assay to monitor the clinical trajectories of four patients with metastatic melanoma. Our report highlights potential benefits and limitations using ctDNA in this setting to inform clinical decision-making. This report provides a proof of concept of the technique using an mPCR-NGS-based ctDNA assay (Signatera ^TM^) in the clinical context and in adjunct with other radiological information. Large cohort prospective trials would be needed to validate the utility and validity of this approach.

## Introduction

Melanoma is the deadliest form of skin cancer, with an incidence that has continued to increase over the past several decades (1). The introduction of novel treatments, however, has contributed to the substantial improvement in patient outcomes, with an approximately 5% decline in the mortality rate since 2013 (2).

With the integration of paradigm-shifting therapies, including immune checkpoint inhibitors (ICIs) and BRAF-MEK inhibitors, clinical outcomes have improved significantly for patients with advanced melanoma. However, several challenges have also emerged. Pseudoprogression is a well-recognized radiological phenomenon that can occur during treatment with ICI, due to immune cell infiltration in the tumor microenvironment. This phenomenon has been observed to occur in up to one-third of melanoma patients treated with ICI (3). It is difficult to distinguish between true progression and pseudoprogression using standard-of-care imaging, and misinterpretation of radiological findings can adversely affect optimal clinical decision-making. New assessment tools have been developed to evaluate response to ICI (iRECIST), but their use remains limited in the context of clinical trials. Moreover, utilizing imaging as a single modality for surveillance has inherent disadvantages, such as the lack of ability to capture early response to therapy, the early stages of progression, or to monitor disease status over short intervals (4). As such, the identification and validation of novel biomarkers that can accurately monitor disease status and assess treatment efficacy in real time is critically needed.

Circulating tumor DNA (ctDNA) has emerged as a biomarker that has been shown to accurately reflect disease burden in real time. Testing relies on the detection of tumor-related DNA fragments (140–170 base pairs long) in plasma collected from peripheral blood (5). It has been used successfully in many cancer indications for disease screening, disease monitoring, prognostication, and treatment evaluation (6). Multiple platforms have been developed to analyze ctDNA in different cancers including single mutation detection by droplet-digital PCR (ddPCR), next-generation sequencing (NGS), or whole-exome sequencing (WES), which have been reviewed previously (7, 8). Existing evidence on the clinical utility of ctDNA testing in melanoma has emerged recently in retrospective and prospective studies (9, 10). The use of personalized and tumor-informed ctDNA testing has been shown in several studies to detect molecular residual disease (MRD), and serve as an important prognostic tool to predict relapse in colorectal, breast, lung and urothelial cancers (11–14).

Here, we present a case series of four patients with metastatic melanoma treated with different modalities, demonstrating our experience using personalized and tumor-informed testing for longitudinal monitoring of ctDNA, and how ctDNA results could inform clinical decision-making. We also discuss the challenges of using such an assay in the management of patients with metastatic melanoma.

## Methods and case presentation

### Methods

ctDNA was detected and quantified using multiplex (m)PCR-next generation sequencing (NGS)-based ctDNA assay based ctDNA assay (Signatera™), which has been previously described in detail (11). Plasma samples were collected periodically in an alternating fashion with radiological imaging. Due to the retrospective nature of data collection, there were no fixed interval time points at which patients had ctDNA testing performed. We collected two tubes of whole blood (~20 ml) in Streck Cell-Free DNA BCTs for each patient at each time point. All blood samples had plasma isolated within 9 days of collection by single-spin centrifugation of the blood at 22°C, for 30 min at 3,220*g*, and was stored at 4°C until further use. Cell-free DNA extraction from plasma samples was performed using QIAsymphony DSP Circulating DNA Kit. Tumor tissue was collected from all patients as fresh frozen or as formalin-fixed and paraffin-embedded tissue (FFPE). Cell-free DNA was extracted using the Puregene DNA purification kit (Gentra Systems) or using the QiAmp DNA FFPE tissue kit (Qiagen). To design the ctDNA assay for each patient, WES was performed on biopsied tumor tissue, along with a matched-normal whole blood sample. Sequencing results were analyzed using Natera’s proprietary tissue variant calling pipeline, and 16 highly ranked tumor-specific somatic, clonal, single-nucleotide variants (SNVs) were selected for mPCR primer design for each personalized ctDNA assay. Plasma samples were later collected and were subjected to cfDNA extraction, followed by cfDNA library preparation. The cfDNA was end-repaired, A-tailed, and ligated with custom adapters, followed by amplification and purified using Ampure XP beads (Agencourt/Beckman Coulter). A proprietary mPCR methodology was used to run patient-specific assays. The mPCR product is then barcoded, pooled, and sequenced on the Illumina HiSeq 2500. A plasma sample was considered to be ctDNA-positive if at least two out of the 16 SNVs were detected. ctDNA levels were quantified in mean tumor molecules per milliliter of plasma (MTM/ml) (13).

### Case 1

**Figure 1:**
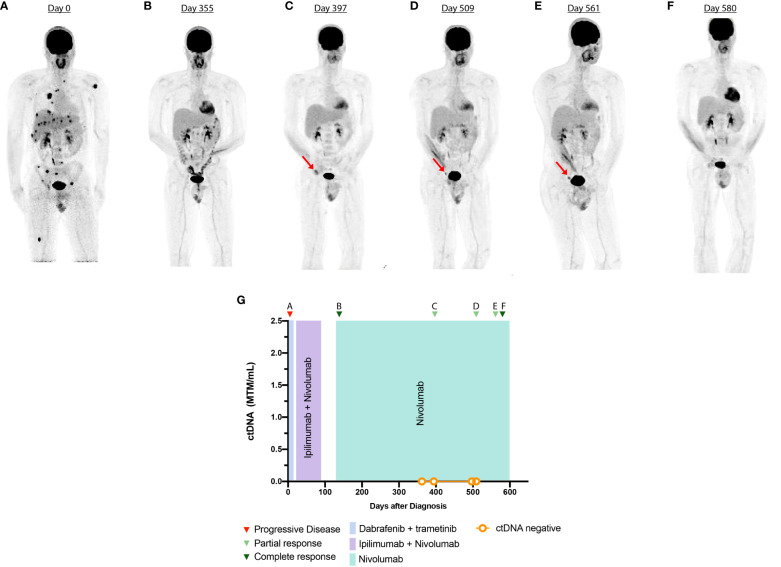
Timeline and imaging of case 1. **(A)** Anterior volume-rendered maximum activity-projection FDG-PET images show intense FDG uptake in the bones, lung, and liver prior to starting immunotherapy (ICI). **(B)** PET demonstrates complete response after three cycles of ICI. **(C–E)** PET demonstrates persistent 2.8 × 1 cm right external iliac lymph node with interval increase in FDG uptake with a maximum SUV of 6. **(F)** PET shows resolution of external iliac lymph node. **(G)** Timeline of administered systemic therapy and ctDNA changes during surveillance.

A 24-year-old man presented with an enlarging abdominal wall skin lesion. Biopsy demonstrated polypoid superficial spreading malignant melanoma. The patient underwent a wide local excision. Biopsy of the left inguinal lymph node was negative for metastatic melanoma. His disease was stage IB (T2aN0M0), and was followed with close dermatologic monitoring. Two and half years later, the patient noticed an enlarged right inguinal lymph node. Excisional biopsy of the right inguinal lymph node demonstrated complete occupancy by metastatic melanoma. A staging PET-CT was performed, which showed widespread metastatic disease (**Figure 1A**). Further staging workup with a brain MRI did not demonstrate intracranial metastases.

Tumor-specific mutational analysis revealed the presence of the *BRAF^V600E^* mutation (**Supplementary Table 1**). As such, the patient was initiated on dabrafenib (150 mg, PO, twice daily) and trametinib (2 mg, PO daily). At this time, he presented to our tertiary academic center for a second opinion. After a discussion of risk and benefits, the patient switched to ipilimumab (3 mg/kg) and nivolumab (1 mg/kg). After three cycles, the patient developed immune-related adverse effects (IRAEs) with grade 1 rash, grade 1 hepatitis, and grade 3 colitis, which was treated with prednisone (1 mg/kg) followed by a slow taper. Imaging at this time showed complete response to the ICI regimen (**Figure 1B**). Ipilimumab was permanently discontinued, and maintenance nivolumab (480 mg, IV every 4 weeks) was continued after resolution of IRAE symptoms. During ICI treatment, an interval PET-CT showed disease response, and ctDNA analysis was negative, showing 0 MTM/ml. However, 10 months after ICI initiation, a restaging PET-CT scan showed increased metabolic activity in the right external iliac lymph node, concerning for disease recurrence (**Figure 1C**). Repeat imaging 2 months later showed interval stability of the size and metabolic activity of an inguinal lymph node (**Figure 1D**). ctDNA analysis at this time was once again negative. A repeat scan 6 months after the initial concern for progression showed resolution of the metabolic activity of the right iliac lymph node (**Figure 1E**). Subsequent scans have demonstrated a complete metabolic response (**Figure 1F**). Treatment with monotherapy nivolumab was continued throughout the follow-up period.

### Case 2

**Figure 2:**
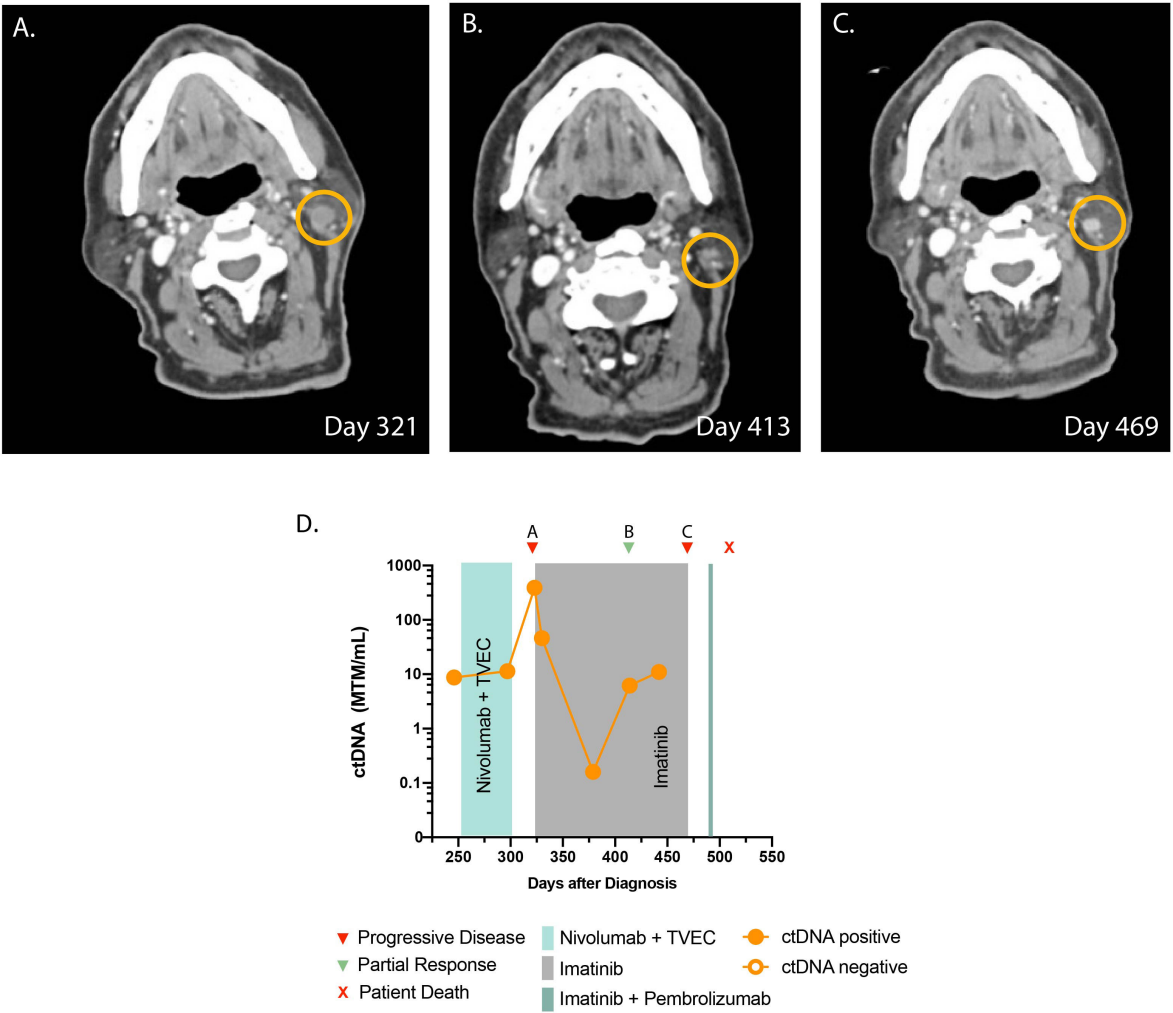
Timeline and imaging of case 2. **(A)** Cross-sectional CT demonstrates progression of infiltrative lesions in a left level 2B lymph node prior to starting imatinib. **(B)** CT demonstrates a decrease in the size of the lesion after starting imatinib. **(C)** CT demonstrates progressive disease. **(D)** Timeline of administered systemic therapy and ctDNA changes (no ctDNA is available beyond day 450 as the patient opted not to have the test).

A 78-year-old man presented with a firm progressive mass on his left scalp. Biopsy showed melanoma and NGS was negative for any actionable mutations. Staging imaging was negative for metastatic disease. The patient underwent a wide local excision with full thickness skin graft and was found to have melanoma, stage IIC (pT4bcN0cM0). At this time, he was started on adjuvant nivolumab. After 5 months, the patient developed three pigmented cutaneous nodules around the surgical graft site, which demonstrated involvement with metastatic melanoma on biopsy. ctDNA testing results were obtained at this time, which indicated low values but detectable at 8.73 MTM/ml (**Figure 2D**). The patient was started on talimogene laherparepvec (TVEC) for the skin lesions intradermally, and nivolumab was continued as there was no evidence of visceral disease progression. However, after four cycles of the therapy, restaging scans showed new pulmonary and pleural metastases. Brain MRI was negative for intracranial disease but a left retroauricular soft tissue lesion was observed (**Figure 2A**). At this time, ctDNA was significantly elevated at 389 MTM/ml (**Figure 2D**). NGS on newly obtained biopsy demonstrated a *KIT* mutation in exon 13 (pK642E) in 2.7% of the variant allele fraction (**Supplementary Table 1**), and the patient started imatinib at 400 mg daily as an off-label indication. After 1 week, his ctDNA decreased to 45.96 MTM/ml, and at 2 months follow-up, there was notable reduction in the size of the retroauricular lesion (**Figure 2B**). Longitudinal ctDNA monitoring showed gradual increase in ctDNA levels. Restaging images at 5 months showed disease progression, which prompted discontinuation of imatinib therapy (**Figure 2C**). He received one dose of pembrolizumab following disease progression. Unfortunately, the patient suffered a catastrophic fall and passed away from issues unrelated to melanoma.

### Case 3

**Figure 3:**
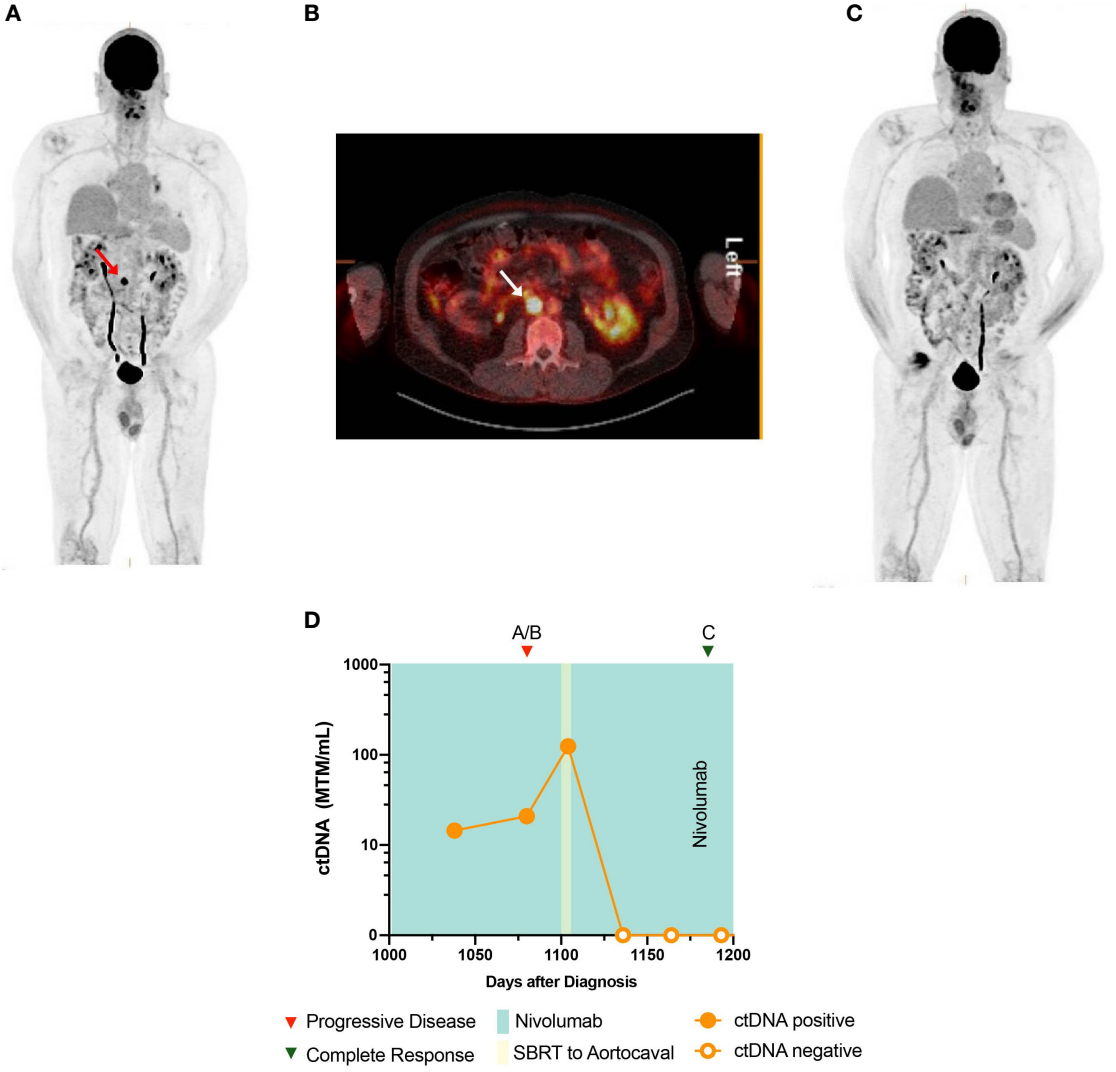
Timeline and imaging of case 3. **(A, B)** PET demonstrates an aortocaval lymph node with a maximum SUV of 21.8. **(C)** PET demonstrates resolution of the aortocaval lymph node after radiation therapy. **(D)** Timeline of case 3 and ctDNA changes.

A 65-year-old man presented with memory difficulties and worsening paresthesias, and was found to have a right frontal heterogeneously enhancing mass. He underwent a craniotomy with gross total resection. Pathology revealed metastatic melanoma with *BRAF^V600E^* mutation (**Supplementary Table 1**). He subsequently underwent hypofractionated stereotactic radiosurgery (SRS) of the resected tumor bed, followed by combination ipilimumab and nivolumab for four cycles. He was then put on maintenance nivolumab. After 4 months, imaging demonstrated recurrent intracranial disease in the right parietal lobe, for which he underwent craniotomy and whole brain radiation. He was subsequently started on BRAF/MEK inhibitors, which were not well tolerated, and therefore, he was switched to nivolumab monotherapy, which was continued for 3 years. Sequential PET-CT showed disease progression in a single aortocaval lymph node measuring 17 × 16 mm with standardized uptake value (SUV) of 21.8 that was not amenable for biopsy (**Figures 3A, B**). At this time, the patient also underwent ctDNA testing, which returned positive, at 20.92 MTM/ml (**Figure 3D**). In response to these disease-positive findings, the patient was treated with stereotactic body radiation therapy (SBRT) (50 Gray in five fractions) for oligometastatic ablation. Near the end of SBRT, his ctDNA levels were markedly elevated at 124.55 MTM/ml. However, subsequent ctDNA measurements after completion of SBRT returned undetectable at 0 MTM/ml, and surveillance imaging showed no evidence of disease (**Figure 3C**).

### Case 4

**Figure 4:**
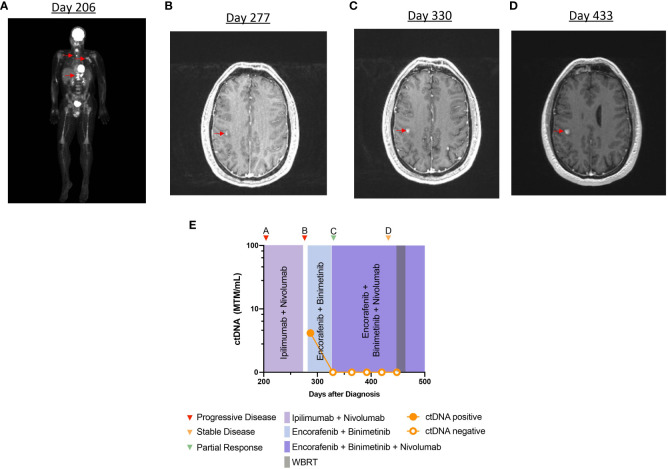
Timeline and imaging of case 4. **(A)** PET scan demonstrates metastatic disease in the bones and lymph nodes. **(B)** Axial section of brain MRI in T1 post contrast phase showing an enhancing parietal lesion. **(C, D)** MRI demonstrates persistent increase in size of enhancing lesions in the brain. **(E)** Timeline of case 4 and clinical course representing treatment sequence and ctDNA changes.

A 31-year-old man with a history of melanoma (pT3aNxMx), stage IIA and positive for *BRAF^V600E^*, was initially monitored with surveillance imaging alone. After 10 months, imaging demonstrated multiple metastases in the thoracic spine, femur, lung, and left axilla (**Figure 4A**). Brain MRI was negative for intracranial disease. The patient underwent vertebroplasty of T11–T12, and he started ipilimumab and nivolumab. Treatment was stopped after the second cycle, due to concern for disease progression. He subsequently switched to dabrafenib (150 mg, PO, twice daily) and trametinib (2 mg, PO, daily), and a partial response to the therapy was observed on imaging. At that time, the patient presented to our center to discuss further management of his melanoma. He elected to restart ipilimumab and nivolumab followed by nivolumab maintenance, with an impression that he did not have true progression during prior ICI. Maintenance nivolumab was continued for 4 months and the patient was noted to have a partial response in extracranial disease to the treatment, on imaging. However, after 4 months of ICI, an MRI of the brain revealed several new intracranial metastases (not all metastases shown on imaging) (**Figure 4B**). The patient was started on encorafenib (450 mg, PO, daily) with binimetinib (45 mg, PO, twice daily). At this time, analysis of ctDNA was 4.16 MTM/ml (**Figure 4E**). Maintenance nivolumab was added to the therapeutic regimen, and repeat imaging after 2 months demonstrated near-complete response in extracranial disease and partial response in the brain metastases (**Figure 4C**). Longitudinal ctDNA monitoring after 4 weeks demonstrated undetectable levels during the treatment period despite continued progression of intracranial metastases (**Figure 4D**).

## Discussion

The emergence of data establishing ctDNA as a predictive and prognostic biomarker to monitor disease status in multiple indications represents a milestone for cancer management. Several platforms have been developed to monitor ctDNA and have been validated in melanoma patients treated with targeted therapy and immunotherapy (15, 16). However, several challenges exist that may hinder the reliability of these tests in clinical practice (17). Technical factors in each specific ctDNA assay are subject to false negativity due to a low amount of tumor DNA allele fraction in isolated plasma as well as low ctDNA shedding from the tumor (17). False positivity represents another challenge that hinders the accurate interpretation of ctDNA, which could arise due to the detection of clonal hematopoiesis of indeterminate significance (CHIP) that is not tumor-specific (18). To this end, personalized, tumor-informed, patient-specific assays of both tumor tissue and matched normal blood samples allow for filtering of CHIP variants, thereby providing an accurate assessment of patient’s tumor burden. The initial analysis of such ctDNA approach is promising and has been validated in several cancer studies, showing high sensitivity ranging from 88% to 100% (11–14). Longitudinal analysis of ctDNA, as measured by ddPCR, was found to be predictive of disease relapse and response to adjuvant treatment in the absence of radiological findings in melanoma (9, 19). Similarly, ctDNA monitoring of melanoma-associated somatic alterations was shown in a cohort of patients to predict disease progression ahead of radiological findings (10). Of note, the majority of the published studies in melanoma have used single mutation or limited targeted panel sequencing for ctDNA analysis (*BRAF*, *NRAS*, *KIT*, and *TERT*) (16, 20, 21). This is of importance, as targeted panel sequencing detects a single, or a limited number of mutations, and its predictive and prognostic value is subject to tumor heterogeneity, as these tests are not able to capture subclonal mutations and new acquired mutations arising during treatment (22, 23). In addition, some mutations common to metastatic melanoma, such as mutations in the *BRAF* gene, can also be detected in patients with benign nevi syndromes (24). Another limitation is the subclonal architecture that could arise during treatment, which cannot be detected with targeted ctDNA panels. These limitations should be considered when interpreting results from panel-based ctDNA assays. Therefore, guidelines do not recommend using ctDNA outside of clinical trials or research given the lack of evidence regarding their utility and validity (25). Of interest, utilizing ctDNA as a supplementary method in the context of clinical, laboratory, and radiological findings might prove beneficial and is an active area of research.

Our experience in this patient cohort demonstrates the clinical utility of ctDNA testing in monitoring response of melanoma to therapy. The ability for ctDNA to detect tumor response to ICI early can aid in distinguishing true progression vs. pseudoprogression. In case 1, ctDNA was used as an adjunctive tool to imaging, to monitor disease response to the therapy. The patient had undetectable levels of ctDNA during treatment with ICI, despite having an abnormal PET-CT scan. The combined approach of short-term imaging with longitudinal ctDNA monitoring helped postpone unnecessary therapeutic intervention, as the FDG avid lesion improved on serial imaging. Lee et al. similarly found that in a cohort of 125 melanoma patients, ctDNA monitoring was able to help differentiate true progression from pseudoprogression during treatment with programmed death-1 (PD-1) inhibitors (26). Therefore, ctDNA testing can potentially reduce the need for unnecessary and invasive procedures normally employed when pseudoprogression is suspected, including repeat biopsy, radiation therapy, and change of treatment (26). The reliability of such an approach for clinical practice will depend on further validation of ctDNA assays in metastatic melanoma, as different detection methods have different sensitivities.

In case 2, we observed a significant decrease in ctDNA levels after starting imatinib, in relapsed metastatic melanoma. The decline in ctDNA occurred prior to the radiological evidence of tumor response to imatinib. In line with this finding, several studies in melanoma demonstrated that ctDNA clearance can precede radiological response during treatment with ICI and targeted therapy (15, 27–32). Case 2 is unique in several ways. Currently, only 30% of relapsed-refractory melanomas respond to KIT inhibitors (1). This case is in concordance with a prior case report demonstrating a possible role for ctDNA in monitoring *KIT*-mutant melanoma response to treatment (31).

Longitudinal monitoring of ctDNA can help clarify radiographic concerns for disease progression that cannot be confirmed by tissue biopsy. This is exemplified in case 3, where elevated ctDNA levels were concordant with a questionable FDG-avid aortocaval lymph node. Interestingly, ctDNA levels increased significantly after starting SBRT for the target lesion prior to ctDNA clearance, consistent with reports that trauma and necrosis due to radiation of tumor cells can lead to increased shedding of ctDNA into the peripheral blood (33, 34). The rise in ctDNA levels prior to clearance confirmed our suspicion that the lesion was secondary to disease progression.

Finally, case 4 highlights the limited ability of ctDNA in metastatic melanoma to monitor response to treatment and disease progression. Immune-privileged sites such as the brain, eye, or testes have highly developed blood–organ barriers that may limit the shedding of ctDNA into the peripheral bloodstream. Several studies have previously shown a poor correlation between ctDNA and intracranial disease status in melanoma (35, 36). In case 4, the patient continued to have undetectable ctDNA levels, despite continued progression of brain metastases. This observation is consistent with the theory that the blood–brain barrier prevents ctDNA shedding. Therefore, ctDNA testing may not be appropriate for monitoring intracranial metastases. Some reports have suggested the potential benefit of using CSF analysis for ctDNA monitoring in this setting. However, this procedure is highly invasive, and needs further validation (37–40).

## Conclusion

In summary, our case series highlights the potential clinical utility and limitations of using patient-informed, tumor-specific ctDNA assay for clinical decision-making in the treatment of metastatic melanoma patients in the real-world setting. Analysis of ctDNA might be beneficial in disease monitoring as a complementary tool added to standard-of-care surveillance methods such as physical examination and imaging. Further validation of the utility of ctDNA in larger melanoma patient cohorts remains essential prior to complete integration of ctDNA testing in the clinical setting.

## Data availability statement

The original contributions presented in the study are included in the article/**Supplementary Material**. Further inquiries can be directed to the corresponding author.

## Ethics statement

Because this work is a case series reporting clinically conducted activities, this activity is not considered to meet federal definitions under the jurisdiction of an IRB and therefore falls outside the purview of the HRPO. IRB ID: 202210030.

## Author contributions

KK and GA conceptualized the idea of the manuscript. KK and AZ performed literature search and wrote the manuscript. KK, GB, and AK conceptualized figures. GB and AK created figures. KK, AZ, OB, GB, AK, and GA reviewed the manuscript and agreed on the final version. All authors contributed to the article and approved the submitted version.

## Acknowledgments

The authors would like to thank Meenakshi Malhotra Ph.D. for reviewing this work.

## Conflict of interest

GB is an employee of Natera and AK is a previous employee of Natera, Inc. Both own stocks in the company.

The remaining authors declare that the research was conducted in the absence of any commercial or financial relationships that could be construed as a potential conflict of interest.

## Publisher’s note

All claims expressed in this article are solely those of the authors and do not necessarily represent those of their affiliated organizations, or those of the publisher, the editors and the reviewers. Any product that may be evaluated in this article, or claim that may be made by its manufacturer, is not guaranteed or endorsed by the publisher.
