# Supplementary material for: Case report: Real-world experience using a personalized cancer-specific circulating tumor DNA assay in different metastatic melanoma scenarios
Source: Front Oncol. 2022 Nov 17;12:978996. doi: 10.3389/fonc.2022.978996 (PMC9713015; doi:10.3389/fonc.2022.978996)
Supplement: Supplementary file 1 [file Table_1.docx]

**Supplementary Table-1: Pathologic mutation detected on next-generation sequencing (NGS) from tumor tissues of patients (TEMPUS assay)**

| **Case Number** | **Pathologic Mutations on NGS (Tempus)** |
| --- | --- |
| **Case 1** | *BRAF* p.V600E Missense variant- GOF  *TERT* c-124C>T Variant- Promoter mutation  *CDKN2A* p.R58* Stop gain- LOF  TMB 3.2 m/MB |
| **Case 2** | *KIT* p.K642E Missense variant (exon 13)- GOF  *GNAS* p.R201C Missense variant- GOF  *ARID1A* p.Q1537* Stop gain- LOF  *B2M* p.E94* Stop gain- LOF  *TP53* p.C242fs Frameshift- LOF  TMB (Not available) |
| **Case 3** | *BRAF* p.V600E Missense variant- GOF  *CDKN2A* p.TR79* Stop gain- LOF  *TERT* c-124C>T Variant- Promoter mutation  *PTEN* Copy number loss  TMB 8.3 m/MB |
| **Case 4** | *BRAF* p.V600E Missense variant- GOF  *TERT* c.-124C>T Variant- Promoter mutation  TMB 4.2 m/MB |

NGS: Next-generation sequencing; GOF: gain of function; LOF: loss of function; TMB: tumor mutation burden
